# Targeting the alternative bile acid synthetic pathway for metabolic diseases

**DOI:** 10.1007/s13238-020-00804-9

**Published:** 2020-11-30

**Authors:** Wei Jia, Meilin Wei, Cynthia Rajani, Xiaojiao Zheng

**Affiliations:** 1 Center for Translational Medicine and Shanghai Key Laboratory of Diabetes Mellitus, Shanghai Jiao Tong University Affiliated Sixth People’s Hospital, Shanghai, 200233 China; 2 School of Chinese Medicine, Hong Kong Baptist University, Kowloon Tong, Hong Kong China; 3 University of Hawaii Cancer Center, Honolulu, HI 96813 USA

**Keywords:** bile acids, gut microbiota, alternative pathway, metabolic diseases

## Abstract

The gut microbiota is profoundly involved in glucose and lipid metabolism, in part by regulating bile acid (BA) metabolism and affecting multiple BA-receptor signaling pathways. BAs are synthesized in the liver by multi-step reactions catalyzed via two distinct routes, the classical pathway (producing the 12α-hydroxylated primary BA, cholic acid), and the alternative pathway (producing the non-12α-hydroxylated primary BA, chenodeoxycholic acid). BA synthesis and excretion is a major pathway of cholesterol and lipid catabolism, and thus, is implicated in a variety of metabolic diseases including obesity, insulin resistance, and nonalcoholic fatty liver disease. Additionally, both oxysterols and BAs function as signaling molecules that activate multiple nuclear and membrane receptor-mediated signaling pathways in various tissues, regulating glucose, lipid homeostasis, inflammation, and energy expenditure. Modulating BA synthesis and composition to regulate BA signaling is an interesting and novel direction for developing therapies for metabolic disease. In this review, we summarize the most recent findings on the role of BA synthetic pathways, with a focus on the role of the alternative pathway, which has been under-investigated, in treating hyperglycemia and fatty liver disease. We also discuss future perspectives to develop promising pharmacological strategies targeting the alternative BA synthetic pathway for the treatment of metabolic diseases.

## INTRODUCTION

The gut microbiota and the host co-metabolize a vast array of small molecule metabolites, many of which play critical roles in shuttling information between the eukaryotic and prokaryotic cells. Many of these small molecule metabolites, such as bile acids (BAs), short-chain fatty acids (SCFAs), and neurotransmitters, are produced and circulated in the gastrointestinal system as well as systemically, and are associated with diverse metabolic disorders including type 2 diabetes mellitus (T2DM), cardiovascular disease (CVD) and non-alcoholic fatty liver disease (NAFLD) (Nicholson et al., 2012; Arora and Bäckhed, 2016; Jia et al., 2018). One example is the contribution of the gut microbiota to the development of chronic liver disease, such as NAFLD, through multiple mechanisms, including increased production of lipopolysaccharides (LPS), inflammatory cytokines (Su, 2002), increased intestinal choline metabolism (Spencer et al., 2011), and dysregulated intestinal and hepatic BA metabolism (Chávez-Talavéra et al., 2017). Hepatic gluconeogenesis is normally suppressed in the presence of increased food-derived glucose in the circulation. However, the accumulation of ectopic triglycerides (TGs) in the liver resulting in insulin resistance, will inhibit the food-derived glucose suppression of gluconeogenesis, leading to the development of hyperglycemia. Therefore, the development of metabolic disorders that involves metabolic interactions among multiple organs including gut, liver, brain, heart and pancreas may necessitate a therapeutic solution that impacts multiple metabolic organs that are mechanistically linked in the disease. Therapeutic targeting of the gut-liver axis in order to reduce liver fat, hepatic gluconeogenesis and lipogenesis for improved glucose and lipid regulation could thus be clinically significant for the treatment of NAFLD and T2DM.

The gut microbiota participates in glucose and lipid metabolism and energy homeostasis, in part by regulating BA metabolism and affecting multiple BA-receptor signaling pathways (Massafra et al., 2018). BAs are steroid acids synthesized in hepatocytes from cholesterol, which are then further modified by gut microbiota. Primary BAs produced in humans are cholic acid (CA) and chenodeoxycholic acid (CDCA), while CA and muricholic acids (MCA) especially, β-MCA, are predominantly produced in rodents. Following the conjugation of the primary BAs to either taurine (predominantly in mice) or glycine (mainly in humans), primary BAs are secreted from the liver into bile and further into the intestinal lumen in response to food ingestion. The intestinal microbiota is capable of biotransforming the intestinal BAs into their unconjugated forms through the action of bile salt hydrolase (BSH). The secondary BAs are then produced through 7α­dehydroxylation or epimerization reactions, notably, deoxycholic acid (DCA) from CA, ursodeoxycholic acid (UDCA) and lithocholic acid (LCA) from CDCA. Muricholic acids, such as α-MCA and β-MCA, are metabolized into ωMCA, hyodeoxycholic acid (HDCA), hyocholic acid (HCA), etc. Most BAs (about 95%) are then reabsorbed in the ileum and transported back to the liver via enterohepatic circulation. The remaining 5%, which escape reabsorption, are excreted in feces (Schaap et al., 2014). BA synthesis and excretion is a major catabolic pathway for cholesterol and lipids. To maintain the BA pool homeostasis, the amount of BAs synthesized in liver must equal to the amount of BA excretion in feces. Thus, inhibition of BA reabsorption increases fecal BA excretion, leading to more BA *de novo* synthesis from cholesterol and attenuation of high-fat-diet induced obesity (Rao et al., 2016).

The membrane G protein-coupled receptor 5, TGR5, and nuclear farnesoid X receptor (FXR), are the two critical receptors of BAs that regulate glucose and lipid metabolism. TGR5 is expressed in brown adipose tissue, muscle tissue, and enteroendocrine cells, where its activation can promote energy expenditure and induce glucagon-like peptide-1 (GLP-1) release to regulate blood glucose levels and attenuate diet-induced obesity (de Aguiar Vallim et al., 2013). FXR is highly expressed in hepatocytes and enterocytes in the distal small intestine and colon. FXR controls several critical metabolic pathways, repressing BA synthesis via the upregulation of ileal fibroblast growth factor 19/15 (*FGF19*/*15*) and hepatic small heterodimer partner (*SHP*), thus maintaining BA homeostasis (Chávez-Talavéra et al., 2017). Meanwhile, suppression of FXR expressed in intestinal L cells induces GLP-1 synthesis (Trabelsi et al., 2015) to promote better glucose homeostasis.

## BA SYNTHESIS: CLASSICAL AND ALTERNATIVE PATHWAYS

BAs are predominantly synthesized in the liver by a number of enzymatic reactions via two different routes. The main pathway which accounts for about 75% of BA production, also called the classical or neutral pathway, is initiated by 7α-hydroxylation of cholesterol catalyzed by the enzyme CYP7A1, followed by further transformations of the steroid nucleus and oxidative cleavage of the side chain involving the enzyme, CYP8B1. The alternative pathway, also called the acidic pathway, is initiated by 27-hydroxylation of cholesterol involving CYP27A1. The oxysterol products of this reaction are further hydroxylated via catalysis by oxysterol 7α-hydroxylase (CYP7B1). The alternative pathway predominantly produces CDCA, and in rodents most CDCA is immediately converted into muricholic acids. Of note, CYP7A1 is the rate-limiting enzyme for BA synthesis and depletion or inhibition of CYP8B1 leads to more BA synthesis via the alternative pathway.

Dysregulation of BA synthesis and metabolism are associated with metabolic disorders in rodents and humans, such as obesity, diabetes, and chronic liver disease (Pandak and Kakiyama, 2019). CYP8B1 is a critical enzyme for CA synthesis, determining the ratio of non-12-OH BAs (CDCA, α/β-MCA, UDCA, LCA, and their conjugates derived from the alternative pathway) to 12-OH BAs (CA, DCA, and their conjugates derived from the classical pathway) (Wahlström et al., 2016). Recently, several studies have reported that depletion or downregulation of CYP8B1 caused a decrease in 12-OH BAs, thus increasing the non-12-OH/12-OH BA ratio resulting in beneficial effects to host metabolic status. *Cyp8b1*^−/−^ mice were found to be resistant to western diet-induced obesity, hepatic steatosis and insulin resistance due to reduction of lipid absorption (Bertaggia et al., 2017). It has also been reported that CYP8B1 deletion in mice improved glucose tolerance by increasing GLP-1 secretion (Kaur et al., 2015). Additionally, depletion of a liver-enriched long non-coding RNA downregulated CYP8B1 expression resulted in an increased conjugated MCA/CA ratio (non-12-OH/12-OH) and enhanced apolipoprotein C2 (ApoC2) expression causing improved lipid metabolism (Li et al., 2015). On the other hand, the expression of CYP8B1 was significantly elevated in diabetic and obese (db/db) mouse liver. Adenovirus-mediated overexpression of *Cyp8b1* increased 12-OH BA levels and induced lipogenic gene expression, including sterol regulatory element-binding protein 1c (*SREBP-1c*), fatty acid synthase (*FAS*) and stearoyl-CoA desaturase 1 (*SCD1*) (Pathak and Chiang, 2019). It has also been found that increased CYP8B1-derived 12-OH BAs were associated with metabolic disorders such as insulin resistance and T2DM in humans (Brufau et al., 2010; Haeusler et al., 2013).

CYP7B1 is another crucial enzyme involved in the alternative BA synthetic pathway. Mice subjected to cold exposure showed metabolic reprogramming leading to enhanced energy expenditure that was partially mediated by CYP7B1. *Cyp7b1*^−/−^ mice significantly downregulated uncoupling protein-1 (UCP-1) expression in BAT, suggesting that CYP7B1 derived BAs might exhibit enhanced TGR5 receptor activation (Worthmann et al., 2017). In diabetes and NAFLD (Biddinger et al., 2008; Chen et al., 2016), reduced hepatic CYP7B1 expression has been reported, suggesting a role of the alternative pathway for metabolic homeostasis in humans. It has also been shown that obese T2DM patients with Roux-en-Y gastric bypass (RYGB) surgery exhibited a higher proportion of serum CDCA before surgery which was correlated with a shorter duration of T2DM (Yu et al., 2015). Higher levels of baseline CDCA were associated with higher rates of diabetes remission after surgery. Therefore, the proportion of CDCA in the total BA pool might act as a potential prognostic marker for the efficacy of RYGB surgery. Thus, it is conceivable that a BA ratio reflecting the interchange of CYP8B1 and CYP7B1 activities may be a key factor determining the homeostasis of glucose and lipid metabolism.

The alteration of BA composition may affect their capacity for emulsification and absorption of dietary lipids (Vaz and Ferdinandusse, 2017). The hydrophilicity of BAs decreases in the order of UDCA > CA > CDCA > DCA > LCA with conjugated BAs being more hydrophilic than free BAs (Monte et al., 2009). BAs produced from the classical pathway such as CA are highly efficient for forming mixed micelles (*ca*. 50 μmol/L) which are necessary for digestion of cholesterol and fat in the intestine. When the BA synthetic pathway shifts to the alternative pathway, more hydrophilic BAs such as UDCA and MCA are produced, resulting in less intestinal cholesterol and fat absorption (Wang et al., 2003). In addition, it has been demonstrated that the 12-OH BA, TDCA, derived from CA, is more likely to increase micelle size and lower the relative hydrophilic-lipophilic balance (HLB) (Matsuoka et al., 2006). Therefore, a “switch” from the classic to the alternative pathway increases the non-12 BA composition, a beneficial effect that contributes to reduced lipid absorption. Moreover, the 12-OH and non-12-OH BAs may have different effects on the rate of BA enterohepatic circulation as well as in the regulation of colonic motility and defecation. Administration of UDCA, a non-12-OH BA, in mice resulted in accelerated BA circulation and was also associated with increased BA fecal excretion, leading to increased production of hepatic BAs and the depletion of hepatic cholesterol (Zhang et al., 2019).

## ACTIVATION OF THE BA ALTERNATIVE PATHWAY RESULTS IN THE IMPROVEMENT OF METABOLISM

**Figure 1:**
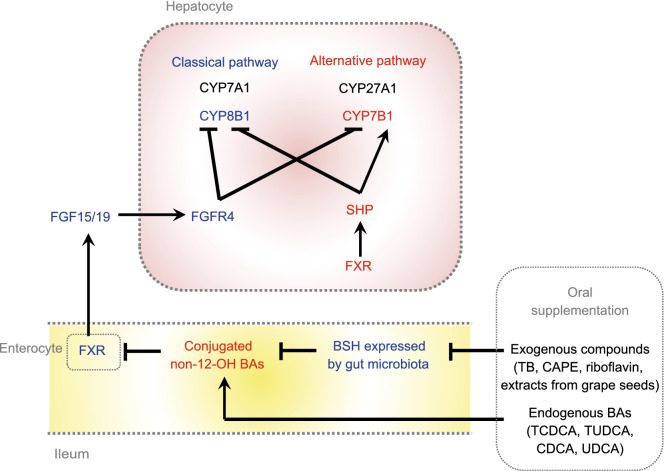
**The mechanism of activation of alternative pathway by exogenous molecules and endogenous BAs.** Oral administration of exogenous molecules suppresses the activity of intestinal BSH enzymes, and causes accumulation of predominantly conjugated non-12-OH BAs. Meanwhile, endogenous conjugated or unconjugated CDCA or UDCA could increase the abundance of non-12-OH BAs directly or indirectly (via enterohepatic circulation). These BAs inhibit intestinal FXR and therefore, downstream FGF15/19-FGFR4 signaling resulting in upregulation of the hepatic BA synthesis genes *CYP7A1*, *CYP8B1*, *CYP27A1* and *CYP7B1* in both classical and alternative pathways. Meanwhile, hepatic FXR expression as well as downstream SHP signaling is increased and the BA synthetic enzymes are inhibited, mainly CYP8B1, in the classical pathway. Ultimately, the combined regulation of intestinal FXR-FGF15 and hepatic FXR-SHP on hepatic BA synthesis results in increased expression of CYP7B1 in the alternative pathway. The metabolites, proteins, and pathways in blue indicate their down-regulation, whereas in red indicate up-regulation after oral treatment of exogenous and endogenous agents

There were several studies published recently, which demonstrated that the activation of the BA alternative pathway had beneficial effects on glucose and lipid metabolism (Fig. 1). Theabrownin (TB) is a polyphenolic compound and a key component of Pu-erh tea with cholesterol- and lipid-lowering effects (Mulder et al., 2001; Huang et al., 2019). Our study showed that oral administration of TB in mice suppressed the activity of intestinal bacterial BSH enzymes. BSH functions to facilitate hydrolysis of conjugated BAs into unconjugated BAs. The suppression of the BSH-positive bacteria resulted in the accumulation of conjugated BAs in the distal ileum. These conjugated BAs, predominantly taurochenodeoxycholic acid (TCDCA) and tauroursodeoxycholic acid (TUDCA), inhibited intestinal FXR and downstream FGF15-FGFR4 signaling and thus, upregulated hepatic expression of the BA synthesis genes *CYP7A1*, *CYP8B1*, *CYP27A1* and *CYP7B1* in both classical and alternative pathways. Meanwhile, increased CDCA production in the liver promoted nuclear FXR expression as well as downstream SHP signaling, and inhibited the BA synthetic enzymes, mainly CYP8B1, in the classical pathway. Ultimately, the combined regulation of intestinal FXR-FGF15 and hepatic FXR-SHP on hepatic BA synthesis resulted in increased expression of CYP7B1 in the alternative pathway, leading to increased production of CDCA rather than CA. Thus, increased BA synthesis decreased cholesterol levels and also increased the excretion of fecal BAs (Huang et al., 2019). This mechanism was further verified by suppression of intestinal FXR signaling using an intestinal-selective FXR agonist, fexaramine (Fang et al., 2015). The increased expression levels of hepatic SHP and CYP7B1 along with the decreased bodyweights, TC, and TG levels induced by TB were reversed after fexaramine administration. When mice were treated with an intestinal-specific FXR antagonist, Z-Guggulsterone, the beneficial effects of TB became stronger. These results confirmed that the cholesterol-lowering effect of TB was due to the inhibition of intestinal FXR signaling resulting from the elevation of hepatic CYP7B1 activity, providing a mechanistic link between intestinal FXR signaling and altered hepatic BA synthesis. Similar to the mechanism of TB regulated gut microbiota, several potent BSH inhibitors, such as riboflavin and caffeic acid phenethyl ester (CAPE), are promising to mediate BA alternative pathway (Smith et al., 2014; Dong and Lee, 2018). Seo et al. (2016) also reported that the extracts from Chardonnay grape seed successfully induced hepatic CYP7B1 expression in mice and reduced hepatic lipids.

UDCA, the first Food and Drug Administration-approved treatment for primary biliary cholangitis, has also been used for the treatment of non-cholestatic, non-hepatobiliary diseases, and non-alcoholic steatohepatitis (Laurin et al. 1996; Lindor et al., 2004; Roma et al., 2011; Mueller et al., 2015). We observed in our study that UDCA supplementation, which led to increased intestinal BA concentrations, predominantly TUDCA and glycoursodeoxycholic acid (GUDCA) (FXR antagonists), induced significantly increased expression of CYP7B1 in the liver. We also found that UDCA administration accelerated the BA enterohepatic circulation rate, which led to both increased BA uptake from the portal vein to the liver and increased BA efflux from the liver to the colon. In this study, FXR acted as a transcription factor for BA synthetic enzymes and transporter proteins, thereby maintaining homeostasis through BA synthesis, influx, and efflux (Mueller et al., 2015). Consistent with our observations, Ma et al. (2017) reported that UDCA supplementation increased CYP7B1 mRNA levels and improved fasting glucose levels and hepatic steatosis in a diet-induced NAFLD model mouse. Our study showed that UDCA administration in mice enhanced CYP7B1 expression but reduced CYP8B1 expression, leading to an elevated non-12-OH/12-OH BA ratio and a decrease in diet-induced obesity. It has been demonstrated that UDCA administration stimulated BA synthesis by reducing intestinal FGF15 secretion, accompanied by elevated serum 7α-hydroxy-4-cholesten-3-one (C4) (Mueller et al., 2015).

Interestingly, oral supplementation of different BAs showed different effects on the BA synthetic pathways. CDCA supplementation in women led to increased BAT activity and glucose uptake accompanied by increased energy expenditure (Broeders et al., 2015). In our studies for TB investigation (Huang et al., 2019), TCDCA or TUDCA treatment in mice induced the expression of hepatic CYP7B1, which resembled the changes induced by TB. However, such metabolic changes were not observed with taurocholic acid (TCA) (12-OH BA) treatment. Taken together, we may conclude that the administration of some BAs belonging to the non-12-OH BAs family would enhance the BA alternative synthetic pathway, thus shaping BA composition.

In summary, the exogenous molecules, such as TB, CAPE, riboflavin, and grape seed extracts act by modifying the gut microbiota so as to reduce BSH activity in the gut. This modification results in increased conjugated BAs, especially TCDCA and TUDCA, in the distal ileum which, in turn, causes inhibition of intestinal FXR signaling. Inhibition of intestinal FXR leads to increased expression of the enzyme CYP7B1, the gatekeeper to the alternative BA synthetic pathway. The overall result is an increased production of CDCA and a shift away from 12-OH-BAs (CA). Increased 12-OH-BAs/non-12-OH-BAs ratios are associated with metabolic disease. Administration of TUDCA, UDCA (which is conjugated to TUDCA in the liver), TCDCA, and CDCA had similar effects on CYP7B1 expression levels. These exogenous molecules or endogenous BAs favor increased production of non-12-OH BAs and improve metabolic phenotypes.

## THE ALTERNATIVE BA SYNTHETIC PATHWAY IS IMPORTANT FOR PRODUCTION OF PHYSIOLOGICALLY IMPORTANT OXYSTEROLS AND FOR DETOXIFICATION OF HARMFUL OXYSTEROLS

The alternative BA synthetic pathway is also the source of very important bioactive lipids, the oxysterols, which are oxidized intermediates in the formation of BAs. In the alternative pathway, cholesterol enters the hepatic mitochondria via the transporter protein, steroidogenic acute regulatory protein-1 (StARD1) where hydroxylation of cholesterol to two important regulatory oxysterols, 25 hydroxy- and 26-hydroxycholesterol (25HC, 26HC, respectively) occurs via the mitochondrial enzyme, CYP27A1 (Pandak and Kakiyama, 2019). The following discussion will center on just these two oxysterols and their metabolites as they relate to liver disease. Oxysterol levels increase in parallel with cell cholesterol content (Pannu et al., 2013). Once formed, oxysterols can then be used as agonists for the liver X receptor (LXR) or they can undergo further transformation to reduce their regulatory capabilities by undergoing a second hydroxylation reaction via CYP7B1 after which most end up in the BA synthetic pathway to produce mainly CDCA (Guillemot-Legris et al., 2016). It should also be pointed out that this pathway not only serves to produce important oxysterols for normal biological functions but serves to then detoxify the oxysterols which are produced in the liver from cholesterol as well as oxysterols from all other tissues in the body (Brown and Jessup, 2009).

Cholesterol removal from peripheral tissues and return to the liver is termed reverse cholesterol transport (RCT). RCT reduces the body’s cholesterol overload (Pandak and Kakiyama, 2019). The oxysterol-dependent activation of LXR causes transcription of multiple genes important for RCT, including the ATP-binding cassette transporters, ABCA1, ABCG1, ABCG5 and ABCG8 as well as apolipoprotein E (ApoE), cholesteryl ester transfer protein, phospholipid transfer protein, scavenger receptor B1 and CYP7A1. Increased expression of CYP7A1 leads to increased synthesis of BAs via the classical pathway and further reduction of cholesterol levels in the body (Pannu et al., 2013). ABCA1 is a cell membrane transporter that facilitates movement of cholesterol and phospholipids from the liver into the plasma onto apolipoprotein-A1 (ApoA-1) to initiate formation of high density lipoprotein (HDL) particles (Oram and Heinecke, 2005). A recent study in rats demonstrated that LXR agonist-induced upregulation of *LXRβ* increased the expression of ABCA1 transporters leading to hypercholesterolemia. Furthermore, in primary biliary cholangitis (PBC) patients, mRNA for LXRβ correlated with the increased levels of ABCA1 mRNA found in liver biopsy samples (Takeyama et al., 2017).

Oxysterol activation of LXR also leads to an increase in *de novo* lipogenesis through LXR induction of carbohydrate response element binding protein (ChREBP) and SREBP-1c which in turn, induce expression of the enzymes necessary for *de novo* lipogenesis, SCD-1, FAS , liver pyruvate kinase (LPK) and acetyl CoA carboxylase-1 (ACC-1) (Pandak and Kakiyama, 2019). Oxysterols also can act in LXR independent ways. They can bind directly to insulin induced gene protein (INSIG) causing it to interact with SREBP cleavage-activating protein (SCAP) to prevent exportation of SREBP-1c to the nucleus and increased lipogenesis thus providing some feedback control (Guillemot-Legris et al., 2016). Oxysterol binding to LXR also mediates glucose homeostasis by decreasing the protein expression of the gluconeogenic enzymes, peroxisome proliferator-activated receptor-γ co-activator-1α, phosphoenolpyruvate carboxykinase (PEPCK) and glucose-6-phosphatase (G6Pase) (Laffitte et al., 2003). Activated LXR also induces increased expression of the insulin-sensitive GLUT4 glucose transporter in adipose and muscle tissue thus improving glucose uptake (Baranowski et al., 2014).

In NAFLD and nonalcoholic steatohepatitis (NASH), changes in cholesterol metabolism and transport occur. Intracellular cholesterol is increased due to reduced expression of CYP7A1 and thus decreased biotransformation of cholesterol to BAs using the classic synthetic pathway (Min et al., 2012). There is also impaired cholesterol efflux from the cell due to decreased ABCA1 activity (Yang et al., 2010) and ABCG6/8 expression (Su et al., 2012). It has been shown that accumulation of free cholesterol (FC) progressively increased with hepatic injury (Min et al., 2012). Hepatic STARD1 protein expression has been shown to be 7- and 15-fold higher than controls for NAFLD and NASH patients, respectively (Caballero et al., 2009). The increase in LXR expression correlated positively with an increase in SREBP-1c and increased lipogenesis leading to increased production of TGs and excess fat storage in the liver (Higuchi et al., 2008). The expression level of CYP7B1, which facilitates the complete bioconversion of oxysterols to BAs, is an important deciding factor for the fate of regulatory oxysterols in the liver in the alternative BA pathway (Kakiyama et al., 2019). CYP7B1 has been demonstrated to be downregulated in NAFLD, NASH without fibrosis and T2DM but becomes up-regulated in NASH with fibrosis which may be due to an upregulation of the alternative BA pathway (Lake et al., 2013; Guillemot-Legris et al., 2016). Insulin resistance has been shown to downregulate expression of Cyp7b1 in the mice with diabetes (Nojima et al., 2013). Serum LXR ligand oxysterols increased in NAFLD patients (Ikegami et al., 2012), and chronically increased oxysterol levels were hypothesized to increase liver inflammation (Clare et al., 1995; Nojima et al., 2013; Guillemot-Legris et al., 2016).

**Figure 2:**
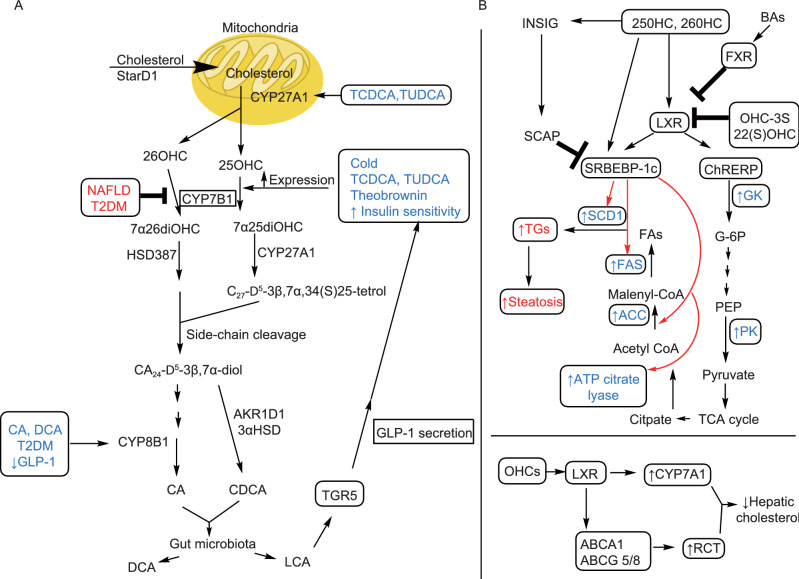
**Diagram of a simplified alternative BA synthetic pathway and its effect on cholesterol metabolism and the importance of oxysterols in lipid metabolism.** (A) Cholesterol is chaperoned into the mitochondria via STARD1 and CYP27A1 converts it to two important bioactive oxysterols, 25OHC and 26OHC. Treatment with the BAs, TCDCA and TUDCA, have been associated with higher expression levels of CYP27A1. The OHCs are then acted on by CYP7B1 to form dihydroxylated species and ultimately, CDCA as the primary product. It is also possible to form CA via CYP8B1 activity in this pathway. In CLD such as T2DM and NAFLD/early NASH, the expression of CYP7B1 is suppressed. Cold temperatures, BA treatment with TCDCA and TUDCA, theobrownin and anything which increases insulin sensitivity induces higher expression of CYP7B1. Decreased GLP-1 (increased insulin resistance), CA and DCA treatment can increase expression of CYP8B1 which has poor patient outcomes. (B) Oxysterols are the first products formed in the alternative BA synthetic pathway and are important endogenous agonists for LXR. LXR activation leads to upregulation of important enzymes in the glycolysis pathway (GK, PK) and also upregulation of enzymes for *de novo* lipogenesis (ATP citrate lyase, ACC, FAS, SCD1). When this pathway becomes overactive as in NAFLD, there is increased fat accumulation in the liver leading to NASH. OHC induced activation of LXR can also cause an increase in RCT as the transporters, ABCA1 and ABCA5/8 are downstream targets of LXR. Activation of LXR also upregulates CYP7A1, the regulatory enzyme that governs BA synthesis in the classical pathway. OHCs can also directly activate INSIG which then forms a complex with SCAP to block the transcription action of SRBEBP-1c. OHCs can also directly activate SRBEBP-1c. BA activation of FXR also acts in an antagonistic way towards LXR activation. The endogenously produced OHC-3S and the synthetically produced 22(S) OHC are also both antagonists to LXR activation. When CYP7B1 is repressed as in insulin resistant metabolic diseases such as CLD, then more cholesterol ends up as oxysterols which in overabundance can cause lipotoxicity and inflammation in the liver

The sulfated form of 25-OHC (25-OHC-3S) has been well studied in mice and human hepatocytes as an LXR antagonist. High-fat diet (HFD) treated mice administered 25-OHC-3S showed decreased body weight, hepatic lipid content and improved glucose tolerance and insulin tolerance relative to untreated HFD mice (Bai et al., 2012; Xu et al., 2013). In human hepatocytes, decreased expression of genes involved in *de novo* lipogenesis (SREBP-1c, ACC and FAS) was observed upon treatment with 25-OHC-3S (Ren et al., 2007). Enhanced expression of PPARγ protein levels and translocation to the nucleus leading to decreased expression of several pro-inflammatory genes has also been reported for 25-OHC-3S treated human macrophages (Xu et al., 2012). 25-OHC-3S can be formed in the cytosol by the sulfotransferase-2B1b (SULT2B1b) (Mutemberezi et al., 2016). The action of mitochondrial CYP11A1 produces 22(R)-OHC, an endogenous oxysterol that has been reported to be another LXR agonist but which is not part of the alternative BA pathway. Interestingly, the exogenous, synthetically produced 22(S)-OHC has been reported to be an LXR antagonist with the effects of decreasing lipogenesis and TG formation in the liver (Guillemot-Legris et al., 2016; Mutemberezi et al., 2016). Another approach to reducing oxysterol activation of LXR is to enhance the expression and activity of CYP7B1. Activation of the G-protein coupled receptor, TGR5 via taurolithocholic acid (TLCA) and LCA which results from gut microbiota metabolism of TCDCA, a product of the alternative BA pathway, stimulates GLP-1 secretion from intestinal endocrine L cells. GLP-1 secretion, in turn, enhances insulin secretion from pancreatic β-cells to improve insulin sensitivity. Improved insulin sensitivity increases the expression of CYP7B1 (Pandak and Kakiyama, 2019). Currently, the strategies mentioned here have not been tested in human trials for chronic liver diseases (CLD) and much has yet to be done to find a way to control the overproduction of oxysterols generated in the alternative BA synthetic pathway and the resulting chronic activation of LXR that can cause the progression of NAFLD to NASH and ultimately, hepatocellular carcinoma (HCC). Figure 2 summarizes the (A) alternative pathway and (B) the oxysterol/LXR axis.

## THE ALTERNATIVE BA SYNTHETIC PATHWAY IS IMPORTANT FOR THE END-STAGE OF NAFLD/NASH, LIVER CANCER

The metabolic disease, NAFLD, can progress to NASH and result in liver cancer. Although not all liver cancer is dependent on formation of NAFLD, we decided to discuss it here in the context of being the final stage of NAFLD/NASH. As discussed in the previous sections, late stage CLD with fibrosis and cirrhosis upregulates the activation of the alternative BA synthetic pathway. This enhancement has been proposed to be a protective, compensatory host response to alter BA composition by producing greater amounts of hydrophilic, non-12-OH-BAs that are more readily excreted by the host (Wang et al., 2003). For enhancement of this pathway to be beneficial in this way, however, depends on the relative and absolute expression level of the key regulatory enzyme in the pathway, an increase of CYP7B1 in activation of the alternative pathway also means an increase in potentially toxic, pro-inflammatory oxysterol levels.

Acyl-CoA acyltransferase 2 (ACAT2) is known to be abundantly expressed in the intestine and in the fetal liver but not in the adult liver (Chang et al., 2000, 2009). ACAT2 has been found to be highly expressed in HCC tumor tissue, is responsible for the synthesis of cholesteryl esters utilizing cholesterol and, more efficiently, oxysterols as a substrate (Chang et al., 2009). Gene expression analysis of 19 paired samples of human HCC and adjacent, non-tumorous samples showed that genes responsible for HDL-sterol influx, cholesterol biosynthesis (*HMGCR*) and sterol sulfonation (*SULT2B1b*) were not significantly different. However, expression of genes involved in sterol catabolism (*CYP8B1*, *CYP27A1*, *CYP7B1*, *CYP39A1*) and sterol efflux (*ABCA1*, *ABCG5*, *ABCG8*) was significantly reduced in HCC tissues implying that sterols accumulate in HCC (Lu et al., 2013). These data also indicate that oxysterol production via the alternative BA synthetic pathway may also be reduced in HCC tumors as the key regulatory enzyme CYP27A1 is reduced (Pandak and Kakiyama, 2019). One could hypothesize that transformed, pre-cancerous cells that evolve in later stages of NASH, usually with the onset of fibrosis and cirrhosis may be responsible in part for signaling the upregulation of the alternative BA synthetic pathway observed for late stage CLD to provide an exogenous source of oxysterols (Lake et al., 2013).

Expression of ACAT2, which directly controls synthesis of cholesteryl esters followed by their incorporation into VLDL was also significantly increased in 50% of the HCC tumors with the highest level of induction observed in more advanced HCC stages along with lack of ACAT2 induction in early stage HCC. In addition, 10 paired samples showed significantly higher amounts of cholesterol and oxysterols in HCC tumor tissue. Furthermore, when ethanol-dissolved oxysterols, 27- and 24-OHC were delivered at high concentration (8-fold higher than physiological plasma concentrations) to HCC cancer cells lines, HepG2 and Huh7, total oxysterols and cholesterol in secreted lipoproteins were significantly increased implying that ACAT2 mediated oxysterol secretion may be protect HCC tumors from excess oxysterol/cholesterol accumulation (Lu et al., 2013).

Oxysterols are important promoters of HCC tumor cell proliferation but at high concentrations (>10 µmol/L) induce apoptosis. Specifically, 25-OHC has been shown to bind to overexpressed endoplasmic reticulum (ER) oxysterol binding protein-related protein 8 (ORP8) in HepG2 and Huh7 cells causing ER stress-induced apoptosis (Li et al., 2016). However, HCC tumors have been shown to have low expression of OPR8, which may protect HCC cells from apoptosis (Zhong et al., 2015).

Elevated *SREBP-1c* mRNA and protein expression have been detected in human HCC tissue when matched to normal tumor-adjacent tissue and was significantly correlated with large tumor size. Analysis of the overall survival (OS) and disease-free (DFS) time in 47 cases for a 3-year follow-up revealed a significant positive correlation between positive expression of SREBP-1c and shorter OS and DFS. *SREBP-1c* knockdown in HepG2 and MHCC97L cells suppressed cell proliferation, migration and invasion (Li et al., 2014). Expression of SREBP-1c is normally controlled by oxysterol activation of LXR but SREBP-1c can also be activated directly by oxysterols in an LXR independent way (Bovenga et al., 2015). It has been reported previously that LXR is highly expressed in HCC tumors, particularly in hepatitis B virus (HBV)-related HCC (Na et al., 2009). In another recent study, however, LXR was found to be significantly lower in HCC tumor tissue relative to adjacent non-cancerous tissue (*n* = 169) and furthermore, LXR expression status associated with tumor stage and metastasis of HCC patients. Higher expression of LXR in HCC patients meant a significantly higher 5-year OS and mean OS than those with low LXR expression, implying that LXR expression status may be useful as a marker for HCC prognosis as well as a potential therapeutic target (Long et al., 2018). Overall, although the increased expression of SREBP-1c has been consistently found in HCC tumor tissue, the fact that its expression does not absolutely require LXR activation but can be activated by oxysterols directly can be used to explain the differences in LXR expression seen in different studies. Therefore the different expression of LXR seen in HCC may be due to other factors as yet undetermined. Thus the role of LXR in tumor tissue remains controversial.

LXR/oxysterol signaling has been reported to create an immunosuppressive microenvironment favoring cancer progression by inhibiting the functional up-regulation of the chemokine (C-C motif) receptor-7 (CCR7) on the surface of maturing dendrocytes (DCs). CCR7 has been shown to be a key receptor that promotes DC location to secondary lymphoid organs where they activate naïve B and T-cells and inhibition of this function thus reduces effective anti-tumor responses (Villablanca et al., 2010; Raccosta et al., 2016). Tumor-released oxysterols can also act in an LXR-independent, (C-X-C motif) chemokine receptor 2 (CXCR2) dependent way to recruit neutrophils within the tumor microenvironment where they act to suppress tumor-specific T-cells and promote neo-angiogenesis (Raccosta et al., 2013, 2016). Here, it can be seen once again that oxysterols can directly act in an immunosuppressive way in HCC tumors and that an immunosuppressive environment can also be generated by oxysterol activation of LXR and therefore the importance of LXR in tumor generated immunosuppression generation remains controversial.

The upregulated alternative BA synthetic pathway observed in late stage liver disease including HCC also leads to increased amounts of CDCA. An observational study of different chronic liver disease stages and changes in serum BAs revealed that patients with cirrhosis plus early stage HCC had higher levels of total BAs with significantly higher levels of conjugated primary BAs, GCA, GCDCA, TCA, TCDCA and TUDCA relative to patients with the same stage of cirrhosis and no HCC (Liu et al., 2019). A recent metabolomics study that compared HCC tissue and serum metabolites revealed CDCA as being significantly increased in both tumor tissue and in serum and was incorporated as a biomarker along with a panel of 5 additional metabolites (Han et al., 2019). The role of elevated CDCA in HCC remains controversial. On the one hand, as previously discussed, the conjugated forms of CDCA and TUDCA act as antagonists of intestinal FXR resulting in inhibition of downstream FGF19-FGFR4 signaling and increased hepatic expression of the BA synthesis genes *CYP7A1*, *CYP8B1*, *CYP27A1* and *CYP7B1* in both classical and alternative pathways. On the other hand, however, increased hepatic CDCA production promoted nuclear FXR expression, downstream SHP signaling, and inhibited the BA synthetic enzyme, CYP8B1, in the classical pathway. The final result of the combined regulation of intestinal FXR-FGF19 and hepatic FXR-SHP resulted in increased expression of CYP7B1 in the alternative pathway, leading to increased production of CDCA rather than CA (Huang et al., 2019). It has been shown that *FGF19* is upregulated in HCC patients and is associated with poor prognosis (Miura et al., 2012; Piglionica et al., 2018). The tumorigenic activity of FGF19 has been shown to be due to crosstalk between the receptor, FGFR4 and β-catenin (Pai et al., 2008; Piglionica et al., 2018). FGF19 is important in the maintenance of BA homeostasis via FXR-mediated CYP7A1 inhibition (Degirolamo et al., 2016). FGF19 is also able to reduce gluconeogenesis, lower TG levels, induce FA oxidation as well as, glycogen and protein synthesis (Fu et al., 2004; Degirolamo et al., 2016). FGF19 has also been shown to protect mice from diet induced obesity (Benoit et al., 2017). Thus inhibition of FGF19 may be a double-edged sword.

In summary, the HCC tumor is heavily dependent on oxysterols for cell proliferation, immune suppression to promote tumor growth, neo-angiogenesis and lipogenesis. The alternative BA synthetic pathway is up-regulated in late-stage liver disease and may be an additional source of oxysterols but may also produce more non-12-OH BAs that are associated with better patient outcomes. The fate of cholesterol in this pathway depends critically on both the absolute and relative expression of CYP7B1. If the enhancement of the pathway produces a greater relative expression of CYP7B1 then more cholesterol will be metabolized to CDCA. If the pathway maintains the same relative expression of CYP7B1 as in NAFLD-NASH stages, then the production of oxysterols to fuel tumor proliferation is more likely. More information and research need to be carried out to find ways to increase the relative expression of CYP7B1 which would increase the value of the alternative BA synthetic pathway in the treatment and prevention of end stage liver disease.

## THE ALTERNATIVE BA SYNTHETIC PATHWAY FOR FETAL LIVER METABOLISM AND GROWTH

The fetal liver (FL) is an important secondary site for maintenance and production of hematopoietic stem cells (HSCs). The fetal liver facilitates the rapid expansion of HSCs while the HSC pool in the bone marrow remains in the quiescent bone marrow niche (Goessling and North, 2016). In a recent study, the rapid proliferation of HSCs in the mouse FL exhibited elevated rates of protein synthesis that was not accompanied by increased ER stress, upregulation of ER chaperones or stress response genes. It was also determined that the FL had a distinct BA profile from the maternal liver governed by the BA synthetic enzymes CYP27A1 present in the highest abundance and CYP8B1. CYP7A1 was found to be expressed lower levels in FL than in the maternal liver (Sigurdsson et al., 2016). It was proposed that the presence of BAs in the FL, although there were no bile duct structures yet developed, inhibited the elevated stress signaling by blocking production of aggregated unfolded proteins (Nakagawa and Setchell, 1990; Itoh and Onishi, 2000; Sigurdsson et al., 2016) Secondary BAs were also found in FL indicating that maternal BAs were freely transported via the placenta into the FL and fetuses from *Cyp27a1*^−/−^ mothers that were *Cyp27a1*^−/−^ had small, underdeveloped livers with markedly decreased presence of BAs indicating the importance of this enzyme for both production of BAs and growth of the FL (Sigurdsson et al., 2016). Despite the increased expression of CYP27A1 in FL, the most abundant BA found was TCA in the HSC study (Sigurdsson et al., 2016) although earlier works have shown TCDCA to be dominant in the FL BA pool (Nakagawa and Setchell, 1990; Itoh and Onishi, 2000)

An example of the latter is the study performed by Itoh and Onishi (2000), on 17 human fetuses whose legal abortion was induced from 13–23 weeks gestation. Some of the important findings were the presence of a decreased ratio of CA/CDCA with increasing gestational age along with a predominance of taurine conjugates. Additionally, the study done by Nakagawa and Setchell (1990) on fetal amniotic fluid revealed BAs that were also detected in early fetal bile (Setchell et al., 1988), including C-1, C-4 (4β-OH-CDCA) and C-6 (hyocholic acid species) hydroxylated species that are not commonly found in adults. During gestation there is also an increase in additional nuclear hydroxylation leading to a progressively more hydrophilic BA composition in fetal bile and amniotic fluid. This was proposed to be a response to the relatively sluggish enterohepatic circulation resulting from immature BA uptake and transport in the fetus (Setchell et al., 1988).

If there is an excess accumulation of BAs in the maternal and fetal plasma, a condition known as intrahepatic cholestasis of pregnancy (ICP) develops that poses increased risk for fetal distress, preterm delivery and even spontaneous fetal death (Wikstrom Shemer et al., 2013; Lofthouse et al., 2019). In ICP, high maternal BA levels result in reverse transfer to the fetus and may also competitively inhibit the transfer of fetal BAs to the mother resulting in the accumulation of maternal BAs in the fetal circulation (Geenes et al., 2014). The treatment for ICP currently involves administering UDCA which has been reported to reduce apoptosis and BA induced oxidative stress and other inflammatory effects on placental trophoblast cells (Roma et al., 2011; Zhang et al., 2016). TCA has been shown to be a vasoactive BA in human placenta capable of raising perfusion pressure in placental cotyledons and constricting chorionic plate arteries causing increased work by the fetal heart to maintain normal placental perfusion. In the same study it was also determined that UDCA was able to block the BA transporter OATP4A1 thus preventing maternal BA uptake across the microvillous membrane of the placental syncytiotrophoblast (Lofthouse et al., 2019).

From the previous sections, UDCA was proposed to enhance the expression of CYP7B1 in the alternative BA synthetic pathway (Zhang et al., 2019). It has also been determined that the FL produces more hydroxylated, hydrophilic BAs than adult livers (Setchell et al., 1988). Another effect of UDCA in ICP could be hypothesized to be to increase hydroxylation of cholesterol in the alternative BA synthetic pathway to produce BA intermediates and BAs that are more readily excreted. The BA synthetic pathway in the FL has yet to be confirmed along the reason for the high expression of CYP27A1 and its dramatic effect on FL growth as well as HSC pool size. A greater understanding of the role of BAs in the FL and their synthesis may help us to understand the effects and the role of the alternative BA synthetic pathway in the adult liver.

## THE RELEVANCE OF BA ALTERNATIVE SYNTHETIC PATHWAY AND GUT MICROBIOTA

**Figure 3:**
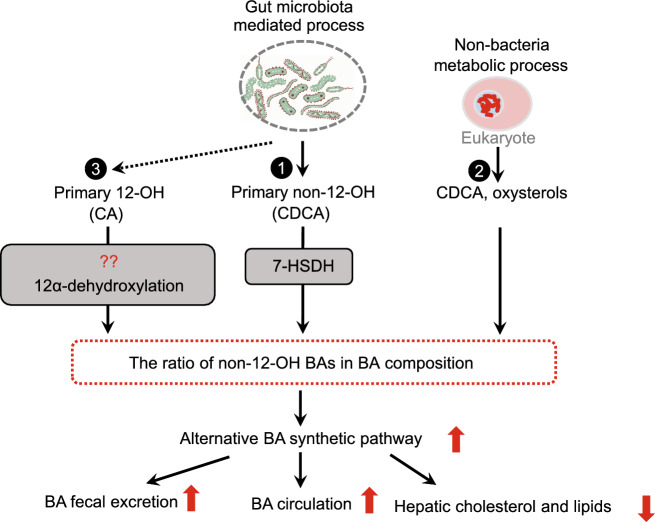
**Alternative BA synthetic pathway manipulated by gut microbiota**. (1) Gut bacteria express 7-hydroxysteroid dehydrogenase (7-HSDH) which catalyzes the epimerization of BA 7-hydroxyl groups and converts the primary non-12-OH BA (CDCA) to UDCA. (2) Non-12-OH BAs can be produced by a non-bacteria metabolic process. (3) Gut bacterial species might also express 12α-dehydroxylase activity, which would convert 12-OH BAs to non-12-OH BAs. The activation of the alternative pathway accelerates BA circulation and fecal excretion and suppresses hepatic cholesterol and lipid metabolism

The alternative pathway of BA synthesis might be manipulated by gut microbiota in addition to being the activated by exogenous CDCA supplementation to increase the BA pool of non-12-OH BAs (Fig. 3).

Gut microbiota has been shown to play a role in hepatic synthesis of BAs with hydroxyl groups at different moieties by altering the BA composition and the ratio of non-12-OH BAs. For example, germ-free (GF) mice are resistant to HFD induced obesity. Compared with conventionally raised (CONV-R) mice, GF mice had reduced FGF15 levels and predominantly increased levels of non-12-OH BAs due to increased CYP7A1 and CYP7B1 but not CYP8B1 expression (Sayin et al., 2013). When the gut microbiota was inhibited in mice by antibiotic administration, FGF15 levels were reduced while the TβMCA/CA ratio was significantly increased, suggesting activation of the alternative BA synthetic pathway. The proportion of TβMCA in the BA pool significantly increased (~80%) in GF mice compared with that in CONR-V mice (50%). On the other hand, injection of FGF15 into wild-type mice significantly suppressed CYP7A1 expression but did not affect that of CYP8B1 (Kim et al., 2007). CYP7A1 is the rate limiting enzyme for BA synthesis, and is also required for CDCA synthesis, due to the crossroad between the classic pathway and alternative pathway. Given that gut microbiota does not regulate CYP8B1 expression (Wahlström et al., 2016), shaping gut microbiota to further regulate intestinal FXR/FGF15 signaling may have greater impact on the alternative BA synthesis pathway. Meanwhile, another possibility could be that reduced FGF15 restriction on BA synthesis robustly enhanced BA synthesis, lead to hepatic BA accumulation causing activation of hepatic FXR signaling and negative feedback control of BA synthesis. Intestinal and hepatic FXR signaling have different regulation effects on BA homeostasis. CYP7A1 is regulated more strongly by the intestinal FXR/FGF15 pathway while CYP8B1 is more sensitive to FXR activation in the liver (Kim et al., 2007). Taken together, we hypothesize that reduced FGF15 levels enhance BA synthesis, mainly via the alternative synthetic pathway, leading to BA composition alterations.

Another possible mechanism for altering BA composition could involve intestinal bacteria, such as the clostridia family, which express 7-HSDH, readily epimerizing 7-hydroxyl groups of BAs in the intestine (Ridlon et al., 2006) and converting CDCA to UDCA. Dihydroxy BAs, such as CDCA and UDCA, are generally better substrates than trihydroxy BAs, such as CA, for these intestinal bacteria (Macdonald et al. 1983; Edenharder et al., 1989). It has been demonstrated that 7-HSDH has the lowest Km and highest V(max) with CDCA and its conjugates (Bennett et al., 2003). Thus, these microbes preferentially metabolize CDCA to UDCA, leading to UDCA accumulation. UDCA species further activate the alternative pathway, accelerate BA circulation, and fecal excretion as we reported (Zhang et al., 2019). We also observed in mouse studies that oral administration of 7-HSDH producing bacteria upregulated the production of alternative pathway derived BAs and enhanced serum C4 levels (Wei et al., 2020). Moreover, BAs, in turn, act to shape the composition of the host gut microbiota. For example, the changes of gut microbiota after CA (the most abundant 12-OH BA in human biliary bile) administration in normal diet-fed mice could resemble those found in high fat diet-fed mice, with an increased firmicutes/bacteroidetes ratio (Yokota et al., 2012). UDCA therapy has been reported to partially restore the altered gut microbial profile in PBC (Tang et al., 2018). Thus, regulation of BA synthesis composition mediated by gut microbiota can further shape the gut microbiota.

## CONCLUSION

In this review, we summarized the role of BA synthetic pathways, producing non-12-OH BAs and oxysterols, which could be signaling molecules that activate multiple nuclear and membrane receptor-mediated signaling pathways in various tissues, regulating the onset and progression of metabolic diseases, such as obesity, T2DM, NAFLD, NASH and even the end-stage of these diseases, liver cancer. The non-12-OH BAs can be derived from dietary supplementation, liver production, and also gut microbiota metabolism. Gut bacteria catalyze the epimerization of BA 7-hydroxyl groups and converts the primary non-12-OH BA (CDCA) to UDCA. Another possible mechanism for the production of non-12-OH BAs is via gut microbial dehydroxylation of 12-OH BAs. However, it is not clear whether gut bacterial species express 12α-dehydroxylase activity, which would convert 12-OH BAs to non-12-OH BAs. Thus far, evidence on the bacterial conversion of 12-OH to non-12-OH BAs is lacking. The role of BA synthesis mediated by gut microbiota-BA crosstalk in the regulation of glucose and lipid metabolism is still emerging. Greater systemic and deeper mechanistic understanding of microbiome dynamics in relation to host system function at a mechanistic level is necessary to leverage new microbiome knowledge for therapeutic purposes. There is a need to identify microbial species that metabolize non-12-OH BAs and thus, affect the ratio of 12OH/non-12-OH BAs. This in turn requires integrative systems modeling, i.e. measurement of dynamic and temporal metabolic variations in relation to organ-organ interactions.

## **ABBREVIATIONS**

ABCA1, ATP-binding cassette A1; ABCG5/8, ATP-binding cassette G5/8; ACC, acetyl CoA carboxylase; BA, bile acid; BSH, bile salt hydrolase; BAT, brown adipose tissue; ChREBP, carbohydrate response element binding protein; CDCA, chenodeoxycholic acid; CA, cholic acid; DCA, deoxycholic acid; FXR, farnesoid X receptor; FAS, fatty acid synthase; GLP-1, glucagon-like peptide-1; OHC, hydroxysterol; INSIG, insulin induced gene; LPS, lipopolysaccharides; LCA, lithocholic acid; LXR, liver X receptor; NAFLD, non-alcoholic fatty liver disease; CYP7B1, oxysterol 7α-hydroxylase; CYP8B1, oxysterol 12α-hydroxylase; CYP7A1, 7α-hydroxylase; RCT, reverse cholesterol transport; RYGB, Roux-en-Y gastric bypass; SCFA, short-chain fatty acid; StARD1, steroidogenic acute regulatory protein; CYP27A1, sterol 27-hydroxylase; SCD1, stearoyl-CoA desaturase; SREBP-1c, sterol regulatory-element-binding protein-1c; TCDCA, taurochenodeoxycholic acid; TUDCA, tauroursodeoxycholic acid; TGs, triglycerides; TB, theabrownin; T2DM, type 2 diabetes mellitus; TGR5, G-protein coupled BA receptor; CLD, chronic liver diseases; NASH, nonalcoholic steatohepatitis; HFD, high-fat diet; GUDCA, glycoursodeoxycholic acid; TCA, taurocholic acid; TLCA, taurolithocholic acid; CVD, cardiovascular disease; MCA, muricholic acids; UDCA, ursodeoxycholic acid; HDCA, hyodeoxycholic acid; HCA, hyocholic acid; SHP, small heterodimer partner; UCP-1, uncoupling protein-1; HLB, hydrophilic-lipophilic balance; FGF19/15, fibroblast growth factor 19/15; CAPE, caffeic acid phenethyl ester; ApoE, apolipoprotein E; ApoA-1, apolipoprotein-A1; HDL, high density lipoprotein; LPK, liver pyruvate kinase; ACC-1, acetyl CoA carboxylase-1; SCAP, SREBP cleavage-activating protein; PGC-1α, peroxisome proliferator-activated receptor-γ coactivator-1α; PEPCK, phosphoenolpyruvate carboxykinase; G6Pase, glucose-6-phosphatase; FC, free cholesterol; SULT2B1b, sulfotransferase-2B1b; ACAT2, acyl-CoA acyltransferase 2; HMGCR, 3-hydroxy-3-methyl-glutaryl-CoA reductase; HCC, hepatocellular carcinoma; ER, endoplasmic reticulum; OS, overall survival; DFS, disease-free survival; DCs, dendrocytes; FL, fetal liver; ICP, intrahepatic cholestasis of pregnancy; CONV-R, conventionally raised; GF, germ-free; 7-HSDH, 7-hydroxysteroid dehydrogenase; PBC, primary biliary cholangitis; C4, 7α-hydroxy-4-cholesten-3-one; TC, total cholesterol; 25HC, 25-hydroxycholesterol; 26HC, 26-hydroxycholesterol; GLUT4, glucose transporter-4; VLDL, very low density lipoprotein; ORP8, oxysterol binding protein-related protein 8; HBV, hepatitis B virus; CCR7, chemokine (C-C motif) receptor-7; CXCR2, (C-X-C motif) chemokine receptor 2; HSCs, hematopoietic stem cells
